# Multicolor Imaging Highlights Xerophthalmic Fundus

**DOI:** 10.18502/jovr.v17i4.12341

**Published:** 2022-11-29

**Authors:** Fatemeh Abdi, Amin Zand, Arzhang Gordiz, Tahmineh Motevasseli

**Affiliations:** ^1^Eye Research Center, The five Senses Institute, Rassoul Akram Hospital, Iran University of Medical Sciences, Tehran, Iran; ^2^Ophthalmic Research Center, Research Institute for Ophthalmology and Vision Science, Shahid Beheshti University of Medical Sciences, Tehran, Iran

##  PRESENTATION

Vitamin A is necessary for the function of retina, by the formation of pigments in rod and cone photoreceptors in the phototransduction cycle. One of the early ocular signs of vitamin A deficiency is nyctalopia. In prolonged untreated cases, other ocular signs including conjunctival Bitot's spots, corneal ulcer, and keratomalacia develop.^[1]^

We present a 30-year-old male who was admitted for evaluation by the rheumatologist due to oligoarthralgia. The patient had bariatric surgery for morbid obesity seven years earlier. The rheumatologist consulted us for the patient's foreign body sensation and night blindness complaints during the last one year.

**Figure 1:**
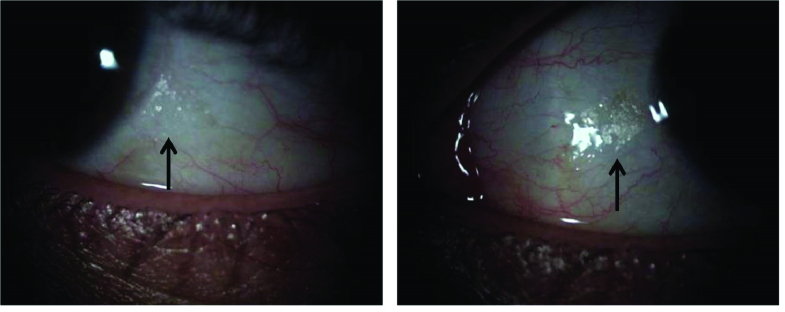
Conjunctival Bitot's spots in the right and left eyes (black arrows).

His ophthalmic examination revealed best-corrected visual acuity of 20/20 in both eyes by Snellen E-chart. Intraocular pressure was 12 mmHg and Ishihara test scores were 14/14 in both eyes. In slit-lamp examination, there was a reduction in tear meniscus height (5 mm) with bilateral Bitot's spots at the limbal conjunctiva [Figure 1].

**Figure 2:**
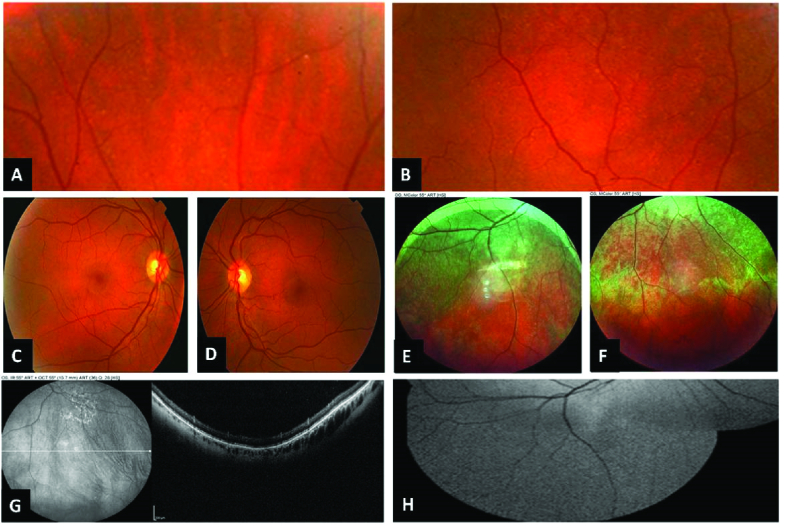
Magnified peripheral fundus photographs of the right (A) and left (B) eyes revealed many small yellowish punctate lesions; optic nerve, macular area, and vessels are normal in the right (C) and left (D) eyes; peripheral fundus multicolor images of the right (E) and left (F) eyes showed much more visible and detectable lesions in comparison to the fundus photographs; peripheral optical coherence tomography detected SDD-like lesions in the left eye (G), right eye revealed similar findings (not shown); magnified fundus autofluorescence showed a mottled hyper- and hypoautofluorescent pattern in the periphery of the retina in the right eye (H), similar to the left eye (not shown).

There were small yellowish punctate lesions in the peripheral retina [Figure 2A–B]. The optic nerve, macula, and vessels seemed normal [Figure 2C–D]. Peripheral spectral-domain optical coherence tomography (SD-OCT) showed sparse subretinal drusenoid deposit (SDD)-like lesions bilaterally [Figure 2G]. Fundus autofluorescence revealed a faint mottled hyper- and hypoautofluorescent pattern in the periphery of the retina [Figure 2H]. In contrast to fundus autofluorescence, multicolor imaging of the peripheral retina detected a vast number of lesions much more visible than in the autofluorescence images [Figure 2E–F] (Spectralis, software version 6.16.6.0; Heidelberg, Germany).

**Figure 3:**
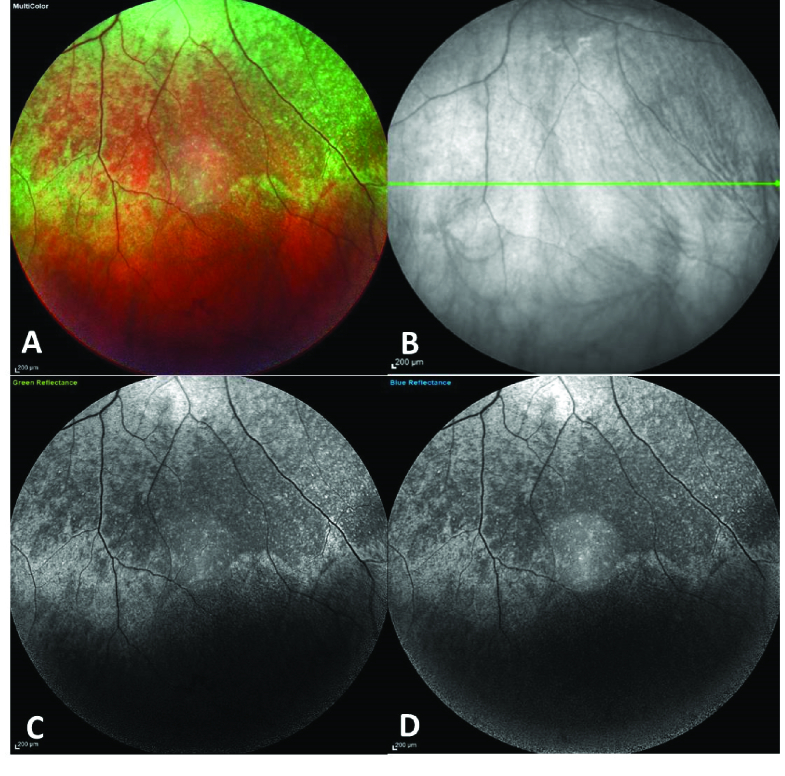
Peripheral fundus multicolor image (A) showed the lesions clearly. The red reflectance did not reveal the lesions (B). Green and blue reflectance showed the same pattern as the multicolor images (C & D).

In multi-color images, when the images from the three different channels were separated to red, green, and blue, we found that the infra-red reflectance photos did not show the lesions, but the blue and green reflectance images highlighted the xerophthalmic lesions similar to the multi-color images [Figure 3].

**Figure 4:**
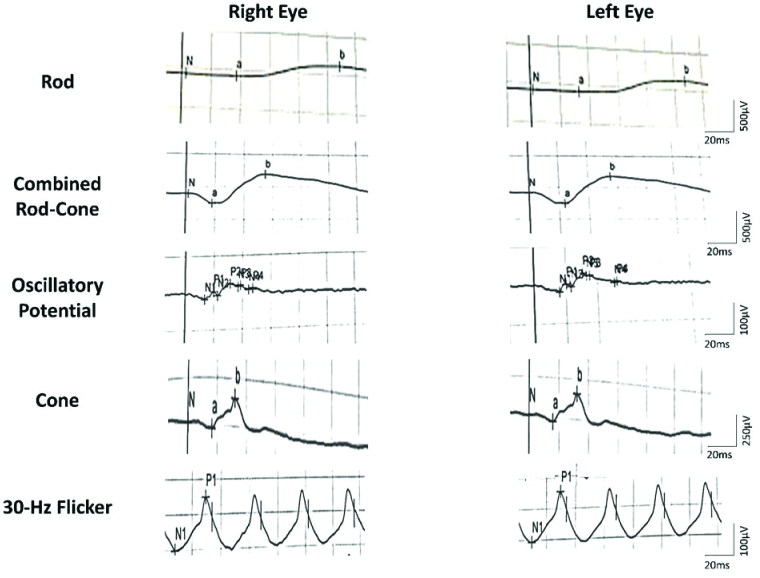
Full-field electroretinography of both eyes detected a reduction in amplitude of rod response and also a smaller reduction in combined rod-cone, oscillatory potential, and cone responses. The amplitude of 30-Hz Flicker responses in both eyes was normal. µV, microvolt; ms, millisecond; Hz, Hertz.

A full-field standard electroretinogram (Ronald Consult, Wiesbaden, Germany) detected a bilateral reduction in amplitude of rod response and also a smaller reduction in combined rod-cone, oscillatory potential, and cone responses [Figure 4]. According to these findings, we suspected vitamin A deficiency. Serum level of vitamin A was 2 μg/dL (normal range: 30–80 μg/dL) and the diagnosis was confirmed.

The patient was treated with intramuscular vitamin A followed by daily administration of oral 50,000 IU of retinol palmitate. The patient did not come back for follow-ups, but upon enquiry, he reported that nyctalopia improved.

##  DISCUSSION

Nowadays, one of the most common causes of vitamin A deficiency is malabsorption after bariatric surgery, like in the case of the current patient.^[2]^

Xerophthalmic fundus lesions as presented in this patient are the accumulation of disrupted photoreceptors' outer segments (especially rods) based on histopathological evaluation.^[3]^ According to previous reports of xerophthalmic fundus, the most common finding in OCT is the disruption of the outer retinal layers, especially the ellipsoid zone and SDD-like lesions in the peripheral retina.^[3,4,5]^ Additionally, multicolor imaging was performed and we found that xerophthalmic retinal lesions were much more detectable by fundus multicolor imaging compared to the infrared reflectance photos. Location of the lesions above the RPE may have caused better visibility of lesions in the green and blue channels. To the best of our knowledge, this is the first report of multicolor imaging in the xerophthalmic fundus.

In summary, acquired nyctalopia needs comprehensive fundus examination and paraclinical evaluations, especially multicolor imaging, to have a better detection of the subtle xerophthalmic fundus changes in vitamin A-deficient patients. Early diagnosis and treatment prevent permanent disruption of photoreceptors and subsequent visual impairment.^[3]^

##  Financial Support and Sponsorship

None.

##  Conflicts of Interest

The authors declare that they have no conflict of interest.
